# Cortical adaptation of the night monkey to a nocturnal niche environment: a comparative non-invasive T1w/T2w myelin study

**DOI:** 10.1007/s00429-022-02591-x

**Published:** 2022-11-18

**Authors:** Takuro Ikeda, Joonas A. Autio, Akihiro Kawasaki, Chiho Takeda, Takayuki Ose, Masahiko Takada, David C. Van Essen, Matthew F. Glasser, Takuya Hayashi

**Affiliations:** 1 Laboratory for Brain Connectomics Imaging, RIKEN Center for Biosystems Dynamics Research, Kobe, Japan; 2 Center for the Evolutionary Origins of Human Behavior, Kyoto University, Inuyama, Japan; 3 Department of Neuroscience, Washington University Medical School, St Louis, MO USA; 4 Department of Radiology, Washington University Medical School, St Louis, MO USA; 5 Department of Brain Connectomics, Kyoto University Graduate School of Medicine, Kyoto, Japan

**Keywords:** Night monkey, Primate, Myelin, Comparative neuroanatomy, Area MT, Auditory cortex

## Abstract

Night monkeys (*Aotus*) are the only genus of monkeys within the Simian lineage that successfully occupy a nocturnal environmental niche. Their behavior is supported by their sensory organs’ distinctive morphological features; however, little is known about their evolutionary adaptations in sensory regions of the cerebral cortex. Here, we investigate this question by exploring the cortical organization of night monkeys using high-resolution in-vivo brain MRI and comparative cortical-surface T1w/T2w myeloarchitectonic mapping. Our results show that the night monkey cerebral cortex has a qualitatively similar but quantitatively different pattern of cortical myelin compared to the diurnal macaque and marmoset monkeys. T1w/T2w myelin and its gradient allowed us to parcellate high myelin areas, including the middle temporal complex (MT +) and auditory cortex, and a low-myelin area, Brodmann area 7 (BA7) in the three species, despite species differences in cortical convolutions. Relative to the total cortical-surface area, those of MT + and the auditory cortex are significantly larger in night monkeys than diurnal monkeys, whereas area BA7 occupies a similar fraction of the cortical sheet in all three species. We propose that the selective expansion of sensory areas dedicated to visual motion and auditory processing in night monkeys may reflect cortical adaptations to a nocturnal environment.

**Supplementary Information:**

The online version contains supplementary material available at 10.1007/s00429-022-02591-x.

## Introduction

Night monkeys, also known as owl monkeys, are distinctive New World primates with a distinctive nocturnal lifestyle (Wright 1989). Phylogenetic studies suggest that, while anthropoids (monkeys, apes, and humans) shifted from nocturnality to diurnality, night monkeys subsequently re-adapted to nocturnality approximately 15–20 million years ago (Hershkovitz 1974; Fleagle 1981; Setoguchi and Rosenberger 1987; Ankel-Simons and Rasmussen 2008). Nocturnal activities require different adaptations from those best suited to a diurnal lifestyle. For example, night monkeys have very large eyes relative to their skull size (thus, the name “owl monkeys”). The large eyeballs and corneas enable increased light gathering on the retina under dim light conditions (Noback 1975). Night monkeys also have a higher density of rod photoreceptors and a lower density of cone photoreceptors in the retina than do diurnal monkeys (Wikler and Rakic 1990), thus exchanging color vision (Jacobs 1977b; Jacobs et al. 1993) for high visual sensitivity under dim light conditions (Jacobs 1977a; Jacobs et al. 1979). Such sensory adaptations of night monkeys may underlie their nocturnal primate niche in the New World (Wright 1989; Warrant 2004); however, little is known about these adaptations at the level of the cerebral cortex.

Over the last half century, many studies on night monkeys have investigated their cortical architecture, connectivity, and function (Allman and Kaas 1971a, b, 1974, 1976; Merzenich et al. 1978; Graham et al. 1979; Baker et al. 1981; Tootell et al. 1985; Kaas 1987, 2004; Malonek et al. 1994; Sereno and Tootell 2005; Sereno et al. 2015). An important early discovery was the middle temporal area (MT) located anterior to areas V1 and V2 (Allman and Kaas 1971b). Subsequent studies suggested that the primary function of MT was motion analysis (Baker et al. 1981; Malonek et al. 1994; Kaskan et al. 2010). Area MT shares common characteristics across various non-human primates (NHP) species, including neural connections (Maunsell and Van Essen 1983; Weller et al. 1984; Krubitzer and Kaas 1990; Palmer and Rosa 2006), architecture (Tootell et al. 1985; Maunsell and van Essen 1987), and receptive field properties (Dubner and Zeki 1971; Van Essen et al. 1981; Baker et al. 1981; Rosa and Elston 1998). Together with the neighboring middle superior temporal (MST) area, MT constitutes the motion processing complex in human (hMT +) (Huk et al. 2002; Kolster et al. 2010; Glasser and Van Essen 2011; Large et al. 2016) and we refer to this region as the MT + complex. Interestingly, a few studies in nocturnal primates reported possible differences in cortical visual systems from those in diurnal primates (e.g., smaller relative size of overall visual cortices, including V1, V2, MT, and MST) (Krubitzer and Kaas 1990; Rosa 2002). It is worth revisiting the issue with modern non-invasive methodology, which could help better understand interspecies differences in structure and function.

Recently, we developed high-quality MRI data acquisition and corticalsurface-based analysis methods, harmonized across primate species including humans (Glasser et al. 2013, 2016), macaques (Donahue et al. 2018; Autio et al. 2020), and marmosets (Hori et al. 2018; Ose et al. 2022) with an aim to establish an improved platform for comparative primate neuroimaging analyses (Van Essen et al. 2019; Autio et al. 2021; Hayashi et al. 2021). This approach has enabled harmonized comparative myeloarchitectonic mapping using the T1w/T2w ratio (Glasser et al. 2014), quantitative comparison of the prefrontal cortex (Donahue et al. 2018), and expansion of the sparsely myelinated association areas in higher primates (Van Essen et al. 2019; Hayashi et al. 2021). As in histological studies that often use myeloarchitecture for parcellating cortical areas, the T1w/T2w ratio myelin in neuroimaging studies aids in parcellation of many cortical areas in humans (Glasser and Van Essen 2011; Glasser et al. 2016) and objective comparisons across species despite very different gyrification patterns (Glasser et al. 2014; Van Essen et al. 2019).

To evaluate evidence pertaining to primate nocturnal adaptation in cerebral cortex, here, we extend our comparative myeloarchitectonic investigation to include night monkeys as well as macaque and marmoset monkeys. Our quantitative interspecies comparison suggests that the relative size of the MT + complex and auditory cortex is significantly larger in night monkeys compared to diurnal primates. This may be associated with evolutionary adaptation of the cerebral cortex to the nocturnal niche environment.

## Methods

### Animals

Ten night monkeys (*Aotus lemurinus*, five males and five females, age = 23.8 ± 6.8 y.o, body weight = 1.08 ± 0.08 kg) were used in this study. All animals were provided by the Center for the Evolutionary Origins of Human Behavior (former Primate Research Institute), Kyoto University (Inuyama, Japan). One monkey (Male, 19.7 y.o, 0.98 kg) with abnormally large ventricles was excluded from the analysis. All experiments were conducted in accordance with the institutional guidelines for animal experiments, Basic Policies for the Conduct of Animals Experiments in Research Institution (MEXT, Japan), and Guidelines for the Care and Use of Laboratory Animals (National Institute of Health, Bethesda, MD). All procedures were approved by the Animal Care and Use Committee of the Kobe Institute of RIKEN (MA2008-03–14).

Animals were initially sedated by intramuscular injection of dexmedetomidine (4.5 µg/kg) and ketamine (6 mg/kg). Anesthesia was maintained with dexmedetomidine (4.5 µg/kg/hr, i.v) and low-dose isoflurane (0.6%, inhalation) in the MRI scanner. Rectal temperature (1030, SA Instruments, Inc. NY, USA) and peripheral oxygen saturation and heart rate (7500FO, NONIN Medical Inc, MN, USA) were continuously monitored.

### Data acquisition

MR scans were carried out using a 3 T MRI scanner (MAGNETOM Prisma, Siemens Healthcare, Erlangen, Germany) and a 24-channel multi-array RF coil designed for scanning non-human primate brains (Rogue Research, Montreal, Canada/Takashima Seisakusho KK, Tokyo, Japan) (Autio et al. 2020). This head coil was originally designed for macaque head size, but proved suitable also for night monkeys. The static magnetic field (B_0_) was shimmed within the brain using the sequence FastestMap (linear projections = 6, averages = 2, volume 25 × 25 × 18 mm, bar FOV = 120 mm, bar thickness = 15 mm, number of echoes = 3) (Gruetter and Tkác 2000). T1w images were acquired using a 3D Magnetization Prepared Rapid Acquisition Gradient Echo (MPRAGE) sequence (0.25 × 0.25 × 0.5 mm^3^, matrix = 512 × 512, slice resolution 50% with interpolation, averages = 3, TR = 2200 ms, TE = 2.2 ms, TI = 900 ms, GRAPPA = 2, bandwidth = 270 Hz/pixel, PE-direction R >  > L, no fat suppression, turbo factor = 176, and pre-scan normalization). T2w images were acquired using a Sampling Perfection with Application optimized Contrast using different angle Evolutions (SPACE) sequence (0.25 × 0.25 × 0.5 mm^3^, matrix = 512 × 512, slice resolution 50% with interpolation, TR = 3000 ms, TE = 562 ms, GRAPPA = 2, bandwidth = 391 Hz/pixel, no fat suppression, turbo factor = 314, and pre-scan normalization). The acquisition time was 18 min and 7 min for scanning T1w and T2w images, respectively.

The B_0_ field-map was estimated using a pair of spin-echo EPI images with opposite phase encoding directions (LR and RL, 1.1 mm isotropic resolution, echo-spacing = 0.95 ms, bandwidth = 1240 Hz/pixel, fat suppression, and pre-scan normalization). The B_0_ field-maps were used for readout distortion correction of T1w and T2w images (Andersson et al. 2003; Glasser et al. 2013).

### Data analysis

#### Image preprocessing

Structural images were pre-processed using a non-human primate (NHP) version of the Human Connectome Project (HCP) pipeline (HCP-NHP pipeline) (Donahue et al. 2018; Autio et al. 2020), FSL (v6.0.4) (Jenkinson et al. 2012), and FreeSurfer v5.3.0-HCP (http://surfer.nmr.mgh.harvard.edu/) (Fischl 2012). The structural preprocessing includes three stages (PreFreeSurferPipeline, FreeSurferPipeline, and PostFreeSurferPipeline), as summarized in Fig. S1. PreFreeSurferPipeline (Fig. S1A) includes registration of T1w and T2w images into an anterior–posterior commissural (ACPC) alignment with a rigid body transformation, brain extraction, correction of B_0_ inhomogeneity-induced distortion, boundary-based registration (Greve and Fischl 2009), and signal intensity correction using bias field estimate (Glasser et al. 2013). The bias-corrected T1w images were registered to a species-specific template rigidly and non-rigidly using linear and nonlinear algorithms in FSL (FLIRT and FNIRT) (Jenkinson et al. 2002). The bias-corrected T2w images were aligned to the T1w images. Both bias-corrected T1w and T2w were upsampled to the 0.25 mm isotropic volumes. To create a species-specific template, T1w and T2w images were aligned and averaged across subjects to generate the standard space NightMonkeyRIKEN-KU9.

The Free Surfer Pipeline was used to reconstruct the cortical surfaces (Fig. S1B). This process started with adjusting the 0.25 mm isotropic NIFTI volume headers of the T1w and T2w to 1 mm isotropic to scale the brain size close to that of humans (Hayashi et al. 2021). Then, intensity correction was applied using FMRIB’s Automated Segmentation Tool (FAST) (Zhang et al. 2001), and the whole brain intensity was scaled with a species-specific factor (80 for night monkey). Following these processes, brain extraction and segmentation of subcortical structures were performed using a Gaussian classifier atlas (GCA) (Fischl et al. 2002), which was created for night monkeys using the current dataset. White matter segmentation was performed based on the segmented subcortical structures (aseg.mgz) plus a white matter skeleton template of night monkey (Hayashi et al. 2021), which fills the thin white matter blades in the anterior temporal and occipital cortex for better surface estimation. White matter surfaces were reconstructed using an HCP-customized *mris_make_surface* in FreeSurfer v5.3.0-HCP. After white matter surface estimation, the surface and volume data were rescaled from the expanded 1.0 mm space back to the 0.25 mm native space. The pial surface was estimated initially using intensity normalized T1w image followed using the T2w image to exclude dura and blood vessels (Glasser et al. 2013; Autio et al. 2020). In the initial T1w-based pial surface estimation process, maximal cortical thickness was 4 mm, and the gray matter threshold was 8 sigma for species-specific optimization.

The Post Free Surfer Pipeline registered individual volume and surface data into those of NightMonkeyRIKEN-KU9 (Fig. S1C). The left and right cortical surfaces generated by FreeSurfer (in ‘native’ mesh) were symmetrized using fs_L-to-fs_LR and fs_R-to-fs_LR surface transformation that was previously generated for the macaque monkey (Van Essen et al. 2012), followed by surface registration to the average sulc of NightMonkeyRIKEN-KU9 using Multimodal Surface Matching (MSM) method (Robinson et al. 2018). Then, surfaces and surface metrics of thickness, curvatures, and sulc were resampled to standardized meshes of 164 k and 32 k vertices. Then the mid-thickness surface was created by averaging white and pial surfaces. Inflated and very inflated surfaces were generated from the mid-thickness surface with species-specific inflation scale parameters (eight for night monkey). Myelin maps were generated by calculating the T1w/T2w ratio weighted toward the mid-thickness (Glasser and Van Essen 2011) using a Gaussian function (FWHM = 1.8 mm, which is optimized for night monkey based on median cortical thickness). To remove bias in the myelin map mostly coming from the B_1_ transmit field, the spatial low frequency (sigma = 5 mm) differences between the individual and a symmetrized myelin template generated from the group average of the nine night monkeys were removed (Glasser and Van Essen 2011; Glasser et al. 2013).

The volumes and surfaces dataset were averaged across subjects. A flatmap was also generated using averaged mid-thickness surfaces in the left and right hemispheres by cutting the calcarine sulcus, ventral part of lateral fissure, and principal dimple. We used CARET5 (v5.64) and HCP Workbench (v1.5.0) for generating the flatmap.

#### Cortical parcellation

Spatial derivatives of T1w/T2w myelin contrast (myelin gradient) and cortical thickness were calculated on the averaged mid-thickness surface of NightMonkeyRIKEN-KU9 dataset, with pre-smoothing (sigma = 0.5 mm). Local peaks in the gradient map indicate the local maxima of change in signal (T1w/T2w myelin contrast or thickness) and represent candidate boundaries between cortical areas (Glasser and Van Essen 2011; Glasser et al. 2016). The borders for the heavily myelinated MT + complex and auditory cortex and for the lightly myelinated parietal cortex were defined using both the intensity and the gradient of T1w/T2w myelin contrast on the NightMonkeyRIKEN-KU9 164 k mesh in each hemisphere separately (MT + complex: high T1w/T2w myelin area in the posterior temporal cortex; auditory cortex: medium-to-high T1w/T2w myelin area in the posterior bank of the lateral fissure and superior temporal gyrus; low-myelin parietal cortex (perhaps corresponding to BA7): low T1w/T2w myelin area in the lateral parietal cortex surrounded by high T1w/T2w myelin areas (MT + complex, auditory cortex, and posterior parietal cortex). We also used cortical mean curvature (folding) and its gradient as a reference to define the border between auditory cortex and retroinsular cortex which both showed medium-to-high T1w/T2w myelin contrasts. The boundary of primary visual cortex (V1) was estimated using the gradients in cortical thickness (lateral side) and T1w/T2w myelin contrast (medial side). The borders were then converted to vertex ROIs on the mid-thickness 164 k surface. The surface ROIs were then resampled to a 32 k mid-thickness surface and then applied to each subject’s 32 k surface. This process relies on the folding-based surface registration across subjects to align cortical areas. Surface areas were computed as the sum of the vertex-wise area on the mid-thickness surface in each ROI in the subject’s anatomical native space. Cortical thickness was estimated as the average of vertex-wise cortical thickness in each ROI. Cortical volume in each ROI was estimated as the sum of the vertex-wise wedge volume calculated using the white and pial surfaces in the subject’s anatomical native space.

#### Interspecies comparisons

For interspecies comparisons, we used macaque monkeys (*Macaca mulatta*, 18 males and 4 females, age = 5.3 ± 1.7 y.o, body weight = 5.20 ± 1.33 kg; *Macaca fascicularis*, 10 males, age = 5.4 ± 2.4 y.o, body weight = 4.51 ± 1.50 kg) and marmoset monkeys (*Callithrix jacchus*, 20 males, age = 5.5 ± 2.8 y.o, body weight = 0.38 ± 0.06 kg). For these species, the harmonized HCP-NHP data acquisition (MAGNETOM Prisma, Siemens, 3 T) and data analyses have been described elsewhere (Table S1) (Autio et al. 2020; Hayashi et al. 2021; Ose et al. 2022). Areal borders were defined using the same procedure as in night monkeys. The interspecies differences in the relative surface area (relative to total cortical area), average thickness, and relative cortical volume (as a fraction of total cortical volume) were tested by two-way analysis of variance (ANOVA) with factors of species (macaque, night monkey, and marmoset) and cortical parcel of interest (MT + complex, auditory cortex, BA7, V1). Species effects for each cortical parcel were analyzed using post hoc t test with Bonferroni correction for multiple comparisons across species and parcels.

## Results

### Bran size and cortical topography, thickness, and myeloarchitecture in night monkey

**Fig. 1:**
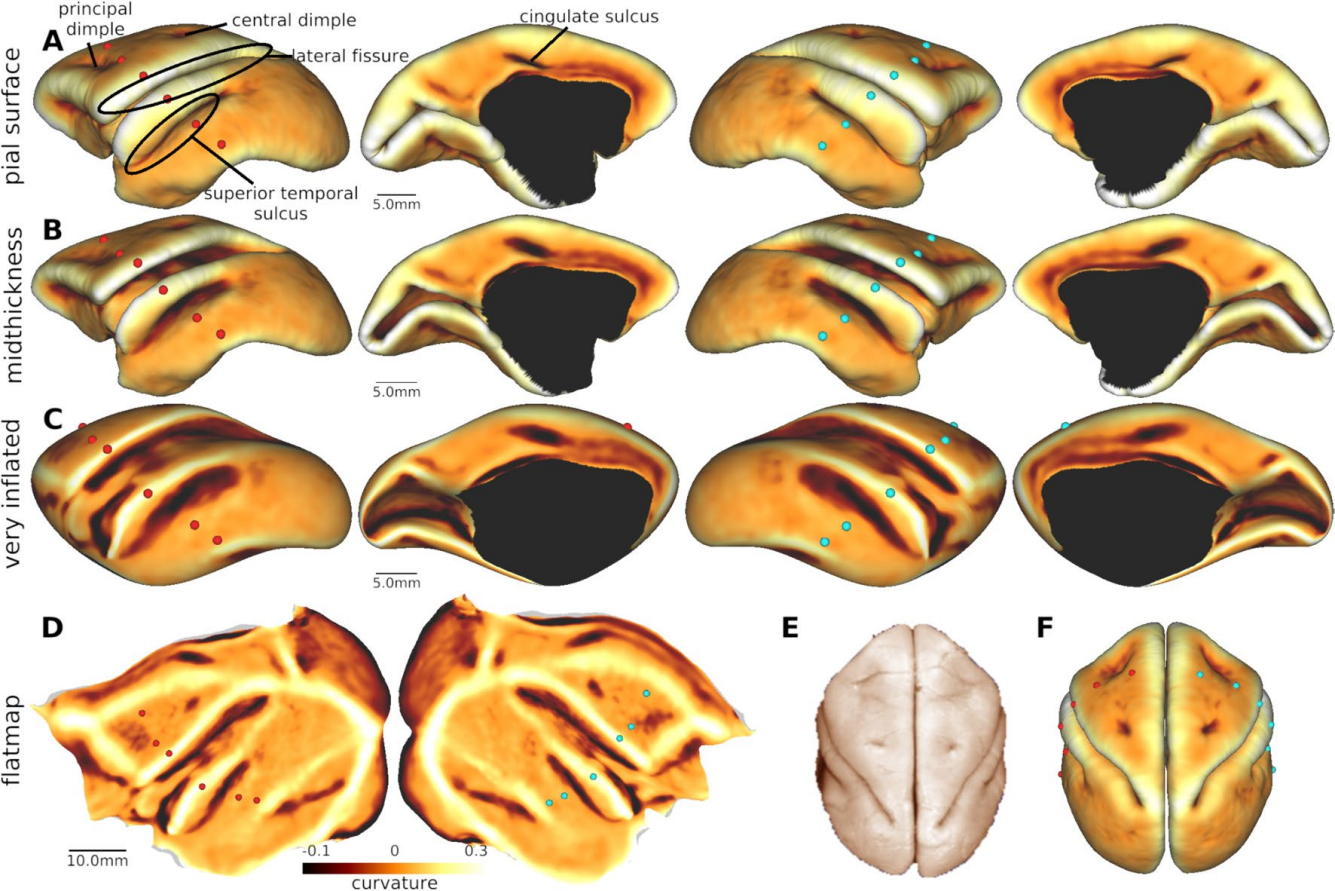
Surface models of night monkey cerebral cortex. Cortical curvature displayed on **A** pial, **B** mid-thickness and **C** very inflated surfaces, and **D** a flatmap. Three sulci (lateral fissure, superior temporal, and cingulate sulcus) and three dimples (principal, arcuate, and central dimple) were consistently identified in all of the animals (*N* = 9). Dorsal views of **(E)** postmortem brain (modified image from http://brainmuseum.org/) and **(F)** reconstructed pial surface. Red dots are placed at regular intervals on the ‘anatomical coordinates’ of the mid-thickness surface. Note that the corresponding red dots are located in a distorted manner in the very-inflated and flat surfaces. The cyan dots in the right hemisphere are vertices with the same ID contralateral to the red dots in the left hemisphere demonstrate symmetrical reconstruction of the cortical surfaces. Dataset is available at https://balsa.wustl.edu/3k7zv

The total volume of the night monkey brain was 18.2 ± 1.2 cm^3^, the volume of cortex (per hemisphere) was 3.73 ± 0.30 cm^3^, and the total surface area of the cortical mid-thickness surface was 20.3 ± 1.3 cm^2^ per hemisphere (Fig. 1A). The cortical pial surface (Fig. 1A) shows a distinct lateral fissure that extends to the dorsoposterior part of the brain, a superior temporal sulcus, a relatively short cingulate sulcus, a central dimple (but no central sulcus), and principal dimple in dorsal prefrontal cortex. These cortical features were consistently observed across all night monkeys. The mid-thickness surface (Fig. 1B), very inflated surface (Fig. 1C), and flatmap (Fig. 1D) also facilitated visualization of large proportions of cerebral cortex buried inside the sulci (e.g., parietal cortex within lateral fissure, medial occipital lobe), albeit with more distorted vertex areas.

**Fig. 2:**
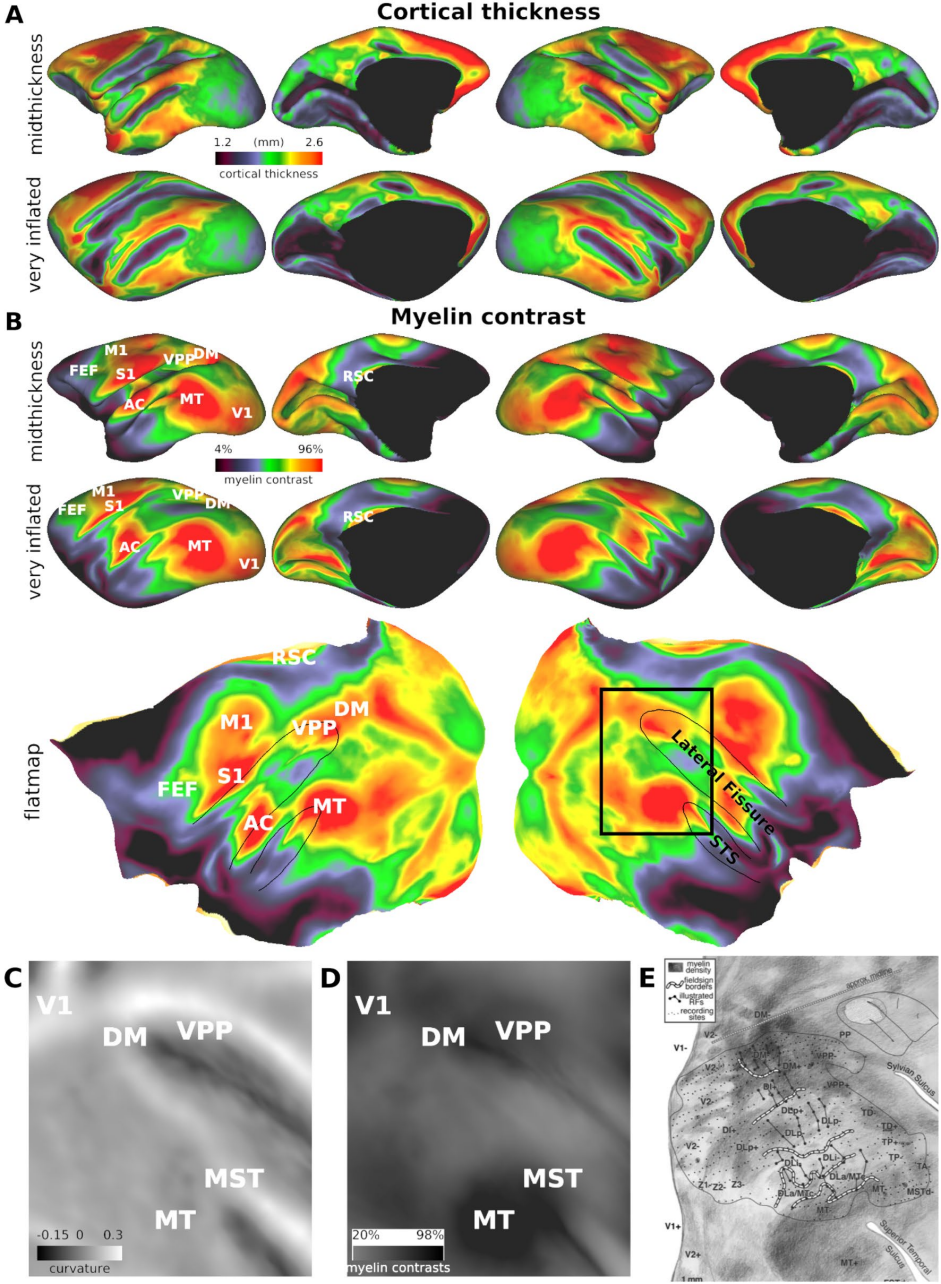
Thickness and myeloarchitecture in the night monkey cerebral cortex. **A** Cortical thickness distribution displayed on mid-thickness (upper) and very inflated surfaces (lower panel). **B** T1w/T2w myelin contrast displayed on mid-thickness (upper panel), very inflated surface (lower panel), and flatmap (lower). The zoomed view of **(C)** curvature and **(D)** T1w/T2w myelin contrast in the parieto-temporal cortical area (the black rectangle in flatmap) in comparison to **(E)** histological flat-mounted section of myelin stain (Sereno et al. 2015). The image intensity indicates myelin density (bright and dark indicate low and high density, respectively). Note the spatial similarity between T1w/T2w myelin contrast and the histological myelin density. Abbreviations: *AC* auditory cortex, *FEF* frontal eye field, *DM* dorsomedial visual area, *MT* middle temporal area, *RSC* retrosplenial cortex, *S1* primary somatosensory cortex, *STS* superior temporal sulcus, *V1* primary visual cortex; *VPP* ventroposterior parietal area. Data at https://balsa.wustl.edu/zK96Z for (**A**) and (**B**) upper panel and https://balsa.wustl.edu/X8qL6 for (**B**) lower panel and (**C**) to (**E**)

The cortical thickness maps shown in Fig. 2A indicate that cerebral cortex is relatively thick in much of prefrontal and lateral parietal cortex, and in both superior and inferior temporal gyri. It is thin in early sensory areas, including occipital cortex, auditory cortex, and somatosensory cortex. The average cortical thickness is 1.91 ± 0.04 mm (*N* = 9), and the lower 5th percentile of cortical thickness in the group average was 1.27 mm. Thus, our image resolution (0.25 × 0.25 × 0.5 mm^3^) was well within the criterion of containing at least two voxels within the thinnest parts of the cortex (Glasser et al. 2016; Autio et al. 2021).

**Fig. 3:**
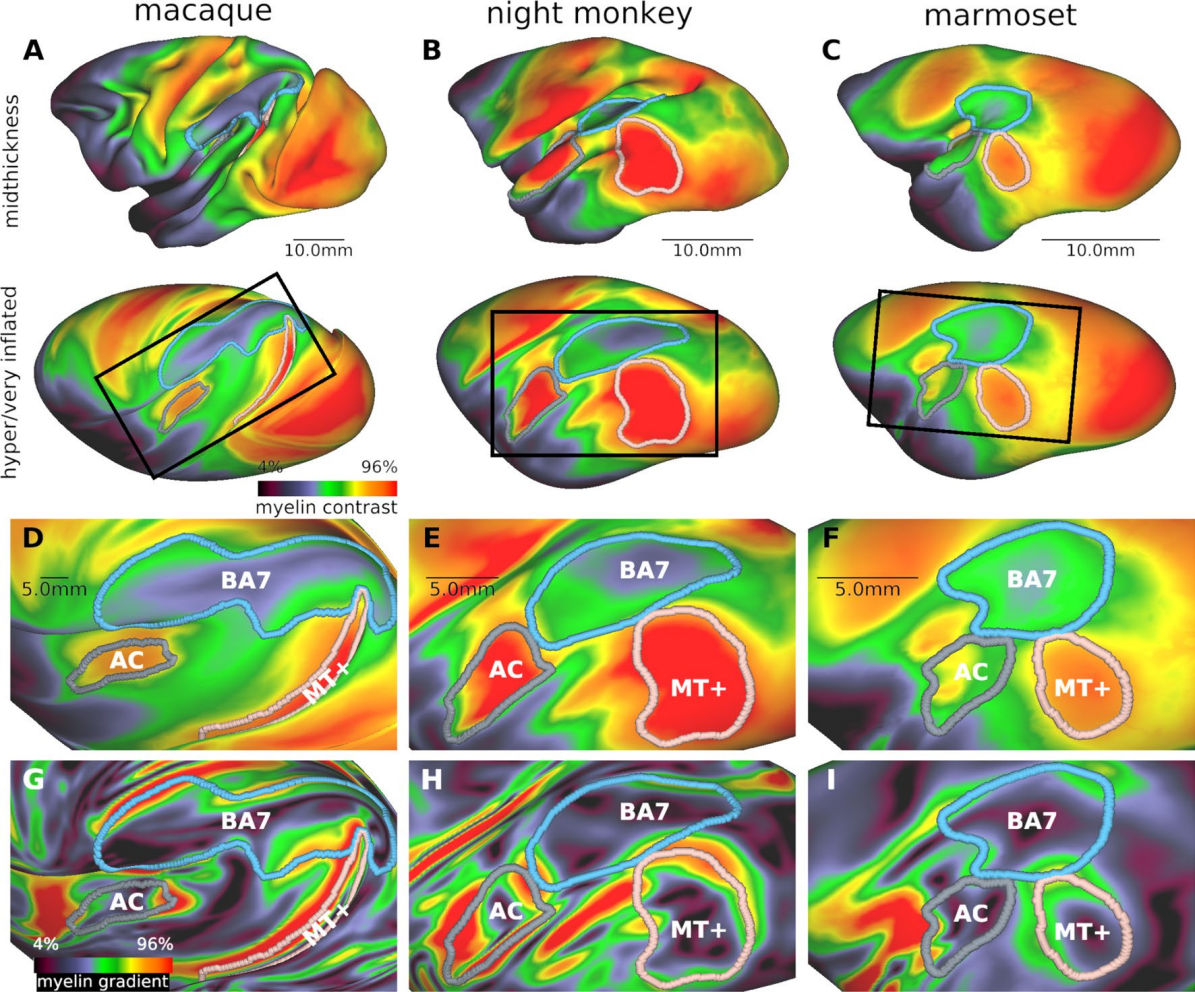
Interspecies comparison of myeloarchitecture in parieto-temporal cortex. T1w/T2w myelin contrast in **A** macaque, **B** night, and **C** marmoset monkeys displayed on a mid-thickness surface (top row) and a hyper inflated **(A)** or very inflated **(B, C)** surface (bottom row). Rectangles indicate the zoomed view of the parieto-temporal cortex in **(D, E, F)** T1w/T2w myelin contrast, and **(G, H, I)** gradient. Note that the shape, topography, and relative area of the MT + complex (MT + ; pink), auditory cortex (AC; gray), and Brodmann area 7 (BA7; cyan) substantially vary across NHP species. Data at https://balsa.wustl.edu/88Pzx

The T1w/T2w myelin maps shown in Fig. 2B show relatively heavy myelination in the primary motor (M1) and somatosensory areas (S1) close to the central dimple, primary auditory (A1), and surrounding auditory cortex, early visual areas, including primary visual cortex (V1), the middle temporal complex (MT +), retrosplenial cortex (RSC), and the dorsomedial (DM) visual area (Fig. 2B). T1w/T2w myelin contrast was moderate in the ventroposterior parietal (VPP) area and frontal eye field (FEF) and relatively low in association areas (e.g., prefrontal, oribitofrontal, medial parietal, insular, and lateral temporal cortices). These trends in T1w/T2w myelin contrast are consistent with other primate species (Glasser et al., 2014; Van Essen et al. 2019; Autio et al. 2020; Hayashi et al. 2021; see Fig. 3 below).

The bottom row of Fig. 2 compares T1w/T2w myelin contrast (Fig. 2D) and histological myelin staining density (Sereno et al. 2015) from a tangentially cut flattened cortex (Fig. 2E). The T1w/T2w map exhibits dense myelination in the MT + complex, DM, and VPP, surrounded by a more lightly myelinated lateral parietal cortex. In particular, the MT + complex (analogous to Sereno and colleagues area MT and MSTd; Fig. 2E) exhibits a sharp transition in histological myelin density relative to the surrounding cortex. The lateral bank of the posterior lateral fissure is lightly myelinated and is surrounded by higher myelin areas such as MT + complex, DM, and VPP (Fig. 2D). Overall, the T1w/T2w myelin contrast (Fig. 2D) and histological myelin density (Fig. 2E) have similar topographic distributions. However, a quantitative validation is hampered by different distortion patterns in the two flatmaps (Fig. 2D, E).

### Interspecies comparison of parieto-temporal cortex

Cortical T1w/T2w myelin contrast, thickness, and their gradients were used to evaluate areal boundaries in macaque, night and marmoset monkeys scanned and pre-processed using the harmonized HCP-NHP methodology (Autio et al. 2020; Hayashi et al. 2021; Ose et al. 2022). In each species, the posterior temporal cortex contained a very heavily myelinated region (Fig. 3A–F, pink border) surrounded by robust gradient-ridges (Fig. 3G–I). This highly myelinated inland likely corresponds to the MT + complex, which includes middle temporal areas MT and MST (Tootell et al. 1985; Desimone and Ungerleider 1986; Large et al. 2016). The medium-to-high T1w/T2w myelin contrast from the posterior bank of lateral fissure to the top of superior temporal gyrus was defined as the auditory cortex in each species (Fig. 3), which adjoins a moderately myelinated retroinsular area located rostromedially (Lewis and Van Essen 2000a). This myelinated area surrounded by strong T1w/T2w myelin gradients likely includes primary auditory cortex (A1) and its surrounding regions such as rostral field (R), caudomedial field (CM), and caudolateral (CL) in night (Imig et al. 1977; Morel and Kaas 1992), macaque (Hackett et al. 1998), and marmoset (de la Mothe et al. 2006) monkeys. Dorso-medial to the MT + complex and auditory cortex, there is an island of relatively low T1w/T2w myelin values (Fig. 3A–F, cyan border) mostly surrounded by robust gradient-ridges (Fig. 3G–I) in each species. In night monkeys, these transitions are supported by histological myelin stain density which also exhibits an island of sparse myelination surrounded by sharp myelin density transitions to the densely myelinated cortex (Fig. 2F). This sparsely myelinated region may correspond to Brodmann area 7 (BA7) complex, which in the macaque includes areas 7a, 7b and 7op and in humans likely even more areas (Yokoyama et al. 2021). We calculated the surface area, average thickness, and cortical volume of these three parieto-temporal parcels for each species along with primary visual cortex (V1), and tested interspecies difference using two-way analysis of variance (ANOVA) with species (macaque, night monkey, marmoset) and cortical parcel (MT + complex, auditory cortex, BA7, V1; see Interspecies comparisons). All variables of relative surface area, average thickness, and relative cortical volume showed significant interaction effect between species and cortical parcel (F_6, 476_ = 2716, 77, 1218, respectively. *p* < 0.001), indicating that patterns of species effects are different among cortical parcels.

**Fig. 4:**
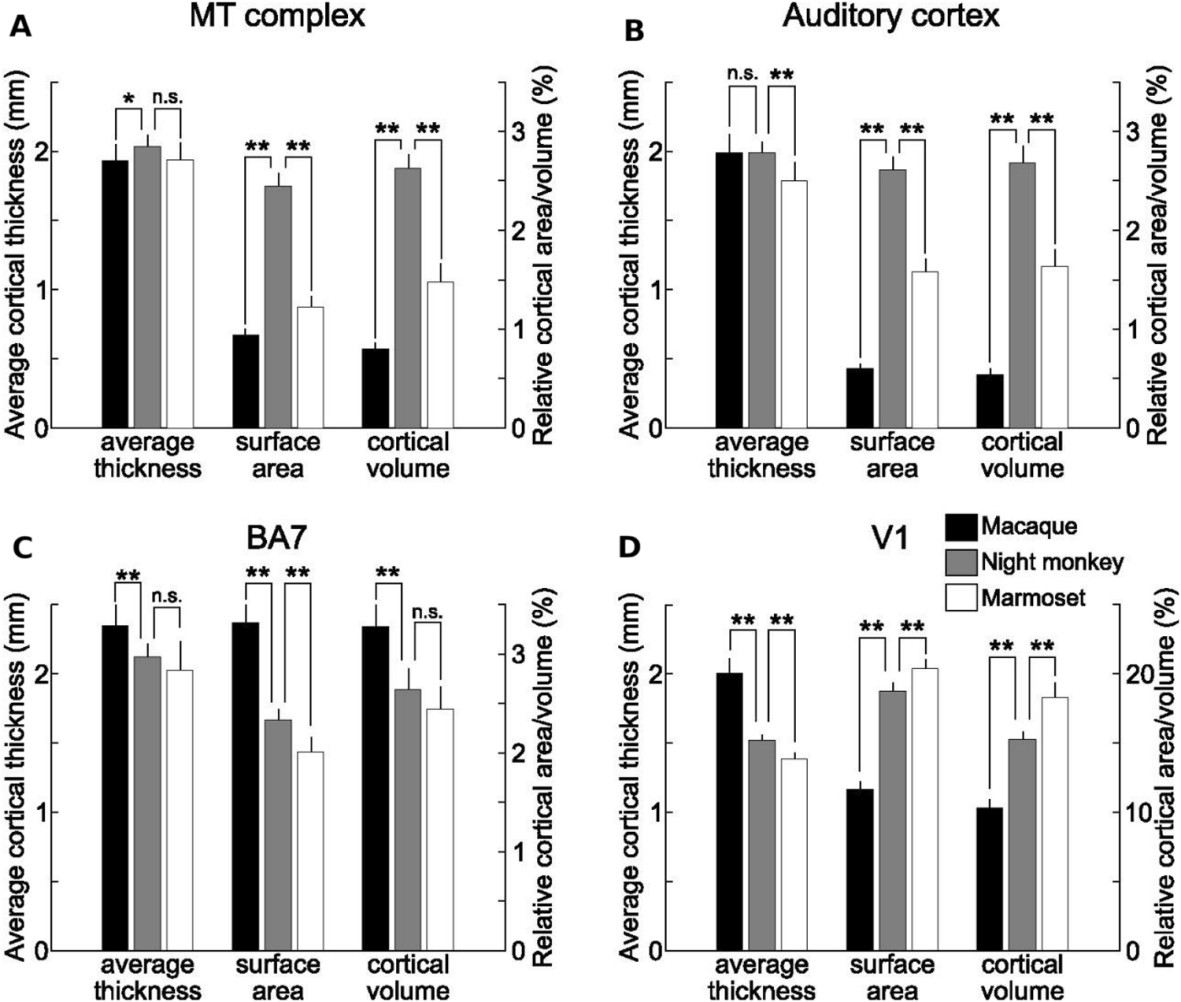
Comparisons of parieto-temporal cortex in non-human primates. Average cortical thickness, surface area relative to the total cortex, and cortical volume relative to the total cortex of MT + complex **(A)**, auditory cortex **(B)**, BA7 **(C)**, and V1 **(D)**. The error bars indicate the standard deviation across subjects (macaque *N* = 32 × 2, night monkey *N* = 9 × 2, marmoset* N* = 20 × 2). Interspecies differences were tested by two-way ANOVA (species and cortical parcels), followed by *t* test with Bonferroni correction; * and ** indicate corrected *p* < 0.05 and 0.001, respectively

The estimated surface area (per hemisphere) of the MT + complex was 89.8, 47.9, and 12.5 mm^2^ in macaque, night monkey, and marmoset, respectively, in reasonable agreement with previous reports (Table 1). Relative to total cortical-surface area, MT + complex was substantially larger in night monkeys (47.9 ± 2.3 mm^2^/2030 ± 128 mm^2^ = 2.4%) in comparison to macaque (89.8 ± 12.8 mm^2^/9894 ± 1470 mm^2^ = 0.9%) and marmoset monkeys (12.5 ± 1.5 mm^2^/1053 ± 55 mm^2^ = 1.2%) (*p* < 0.001 *t* test, Bonferroni corrected) (Fig. 4A). The average cortical thickness of the MT + complex was similar (≈ 2.0 mm) across the three species. Thus, the fractional volume of MT + complex compared to the total volume of cortex is significantly larger in night monkeys (2.6%) in comparison to macaque (0.8%) and marmoset (1.5%) monkeys (*p* < 0.001 *t* test, Bonferroni corrected; see Interspecies comparisons).

**Table 1:** Species comparisons of surface areas of MT + complex, V1, and auditory cortex

Species	Cortical parcel of interest	Surface area (mm^2^) (*N*: number of hemispheres investigated)	Methods	Reference
Macaque	MT + complex/MT (*)	89.8 ± 12.8 (*N* = 64)	T1w/T2w myelin	Current study
		83.1 (*N* = 4)	Myelin staining (modified Heidenhain–Woelke method)	(Gattass and Gross 1981)
		68 (*N* = 1)*	Anterograde neuronal tracing from V1 (^3^H-proline)	(Weller and Kaas 1983)
		76 (*N* = 4)*	Myelin staining (Gallyas or Spielmeyer method)	(Ungerleider and Desimone 1986)
		39 (*N* = 3)*	Myelin staining (Gallyas method)	(Maunsell and van Essen 1987)
		73 (*N* = 10)	Cytochrome oxidase activity	(Sincich et al. 2003)
		78 (*N* = 6)	Myelin staining (Gallyas method)	(Large et al. 2016)
	V1	1156.9 ± 130.7 (*N* = 64)	T1w/T2w myelin	Current study
		1090 (*N* = 1)	Nissl and myelin staining (modified Weigert method)	(Van Essen and Maunsell 1980)
		823 (*N* = 2)	Myelin staining (modified Heidenhain-Woelke method) & recording	(Gattass et al. 1981)
		955 (*N* = 1)	Electrical recording	(Weller and Kaas 1983)
		1195 (*N* = 31)	Electrical recording	(Van Essen et al. 1984)
		1343 (*N* = 11)	Cytochrome oxidase activity	(Sincich et al. 2003)
	Auditory cortex	57.7 ± 11.3 (*N* = 64)	T1w/T2w myelin	Current study
		88 (*N* = 10)	Cytochrome oxidase activity	(Sincich et al. 2003)
Night monkey	MT + complex/MT (*)	47.9 ± 2.3 (*N* = 18)	T1w/T2w myelin	Current study
		37 (*N* = 14)*	Cytochrome oxidase activity	(Tootell et al. 1985)
	V1	381.3 ± 32.2 (*N* = 18)	T1w/T2w myelin	Current study
		286	Electrical recording	(Myerson et al. 1977)
		400 (*N* = 14)	Cytochrome oxidase activity	(Tootell et al. 1985)
	Auditory cortex	51.2 ± 2.9 (*N* = 18)	T1w/T2w myelin	Current study
		48 (*N* = 22)	Nissl and electrical recording	(Imig et al. 1977)
Marmoset	MT + complex	16.1 ± 1.7 (*N* = 40)	T1w/T2w myelin	Current study
		14 (*N* = 6)	Myelin staining (modified Heidenhain–Woelke method)	(Pessoa et al. 1992)
	V1	215.2 ± 13.2 (*N* = 40)	T1w/T2w myelin	Current study
		182 (*N* = 6)	Myelin staining (modified Heidenhain–Woelke method)	(Pessoa et al. 1992)
		194 (*N* = 5)	Nissl staining	(Missler et al. 1993)
		205 (*N* = 4)	Electrical recording	(Fritsches and Rosa 1996)
	Auditory cortex	12.2 ± 1.3 (*N* = 40)	T1w/T2w myelin	Current study
		8–12 (*N* = 5)	Electrical recording	(Aitkin et al. 1986)

Note that the definition of each region varies across studies

*Studies specifically focusing on area MT only. Area estimates using histology may also be underestimated due to brain shrinkage; however, some of the studies compensated for that: 12% (Ungerleider and Desimone 1986), 16% (Van Essen and Maunsell 1980; Van Essen et al. 1984; Maunsell and van Essen 1987; Pessoa et al. 1992), 20–35% (Imig et al. 1977), and unspecified (Fritsches and Rosa 1996).

Auditory cortex showed similar trends with the MT + complex. The estimated surface area (per hemisphere) of the auditory cortex was 57.7, 51.2, and 16.1 mm^2^ in macaque, night monkey, and marmoset, respectively, which are also in good agreement with previous reports (Table 1). The relative surface area of auditory cortex was larger in night monkey (51.2 ± 2.9 mm^2^/2030 ± 128 mm^2^ = 2.5%) than macaque (57.7 ± 11.3 mm^2^/9894 ± 1470 mm^2^ = 0.6%) and marmoset monkeys (16.1 ± 1.7mm^2^/1053 ± 55 mm^2^ = 1.5%) (*p* < 0.01 *t* test, Bonferroni corrected) (Fig. 4B). The average cortical thickness of the auditory cortex was comparable in night and macaque monkeys (≈ 2.0 mm); however, it was slightly thinner in marmosets (≈ 1.8 mm). The fractional volume of auditory cortex relative to the total volume of the cortex was significantly larger in night monkeys (2.7%) in comparison to macaque (0.5%) and marmoset (1.6%) monkeys (*p* < 0.001 *t* test, Bonferroni corrected).

In contrast, BA7 showed a contrasting pattern of interspecies difference. The relative surface area of BA7 was substantially smaller in night monkeys (45.8 ± 2.6 mm^2^/2030 ± 128 mm^2^ = 2.3%) in comparison to macaque (320 ± 54 mm^2^/9894 ± 1470 mm^2^ = 3.2%), but larger than in marmoset monkeys (20.6 ± 2.0 mm^2^/1053 ± 55 mm^2^ = 2.0%) (*p* < 0.01 *t* test, Bonferroni corrected) (Fig. 4C). The average cortical thickness of BA7 was 2.4, 2.2, and 2.1 mm in macaque, night, and marmoset monkeys, respectively. Accordingly, the volume of BA7 relative to the total volume of the cortex was smaller in night monkeys (2.6%) in comparison to macaque (3.3%) (*p* < 0.001 *t* test, Bonferroni corrected).

The different patterns of interspecies effects might simply reflect differences between sensory cortex (MT + complex and auditory cortex) and association cortex (BA7). To control for this, we also compared V1, a heavily myelinated visual area in the occipital cortex (Fig. S2). The estimated surface area (per hemisphere) of V1 was approximately 1160, 380, and 220 mm^2^ in macaque, night monkey, and marmoset, respectively. Although the V1 boundaries were less clear than those of the MT + complex and auditory cortex, our estimates are comparable to previous reports (Table 1, Table S2). The relative surface area of V1 was significantly larger in night monkeys (381 ± 32 mm^2^/2030 ± 128 mm^2^ = 18.8%) in comparison to macaques (1160 ± 181 mm^2^/9894 ± 1470 mm^2^ = 11.7%), but smaller than in marmosets (215 ± 13 mm^2^/1053 ± 55 mm^2^ = 20.4%) (*p* < 0.001 *t* test, Bonferroni corrected) (Fig. 4D). The average cortical thickness of V1 was significantly thinner in night monkeys (1.5 mm) and marmosets (1.4 mm) compared to macaques (2.0 mm). Accordingly, the volume of V1 relative to the total volume of cortex was 10.3, 15.3, and 18.3% in macaques, night, and marmoset monkeys, respectively. Thus, unlike the MT + complex and auditory cortex, the relative surface area/cortical volume of V1 was distinct from all three of the other areas: smallest in macaques and largest in marmosets. These results suggest that the expansion of the MT + complex and auditory cortex in night monkeys is specific to those regions, and not the result of general expansion of sensory/visual areas.

## Discussion

In this study, we have presented an extension of species-harmonized data acquisition and analysis methodology to investigate topography, thickness, and myeloarchitecture of the night monkey cerebral cortex. Our results demonstrated that T1w/T2w myelin contrast in night monkeys is closely associated with histological myelin density in the occipital and parietal areas. Interspecies comparison of cortical myeloarchitecture revealed a similar pattern among NHPs, except that the relative cortical sizes of the MT + complex and auditory cortex in night monkeys were twice as large as those in macaques and marmosets. We propose that this selective visuo-auditory cortical expansion is associated with the nocturnal night monkey’s ecological niche.

### Neurobiological factors of distinct sensory systems in the night monkey

Although there are various nocturnal primate species in Strepsirrhini prosimians, *Aotus* is the only nocturnal monkey genus among Simian primates. Because night monkeys retain foveal structure (Silveira et al. 1993), and lack a *tapetum lucidum* behind the retina (responsible for enhancing illumination under dim light conditions) commonly seen in nocturnal animals (Jones 1965; Martin 1975), they are thought to have re-adapted to nocturnality in a different way from many other nocturnal mammals. The neurobiological factors for adaptation to night vision include enlargement of the eyes (Ross and Kirk 2007), increased maximal pupil diameter (Noback 1975), high rod and low cone retinal density (Ogden 1975; Wikler and Rakic 1990; Silveira et al. 2001), and well-developed magnocellular layer in the lateral geniculate nucleus (Hassler 1966; Diamond et al. 1985). Here, using comparative myeloarchitectonic cortical-surface mapping, we found that the sizes (relative to the total cortical-surface area) of the MT + complex and the auditory cortex were significantly larger in nocturnal night monkeys than in exemplar diurnal NHPs (i.e., macaques and marmosets) (Fig. 4A, C).

The expansion of the MT + complex may support improved motion perception (Petersen et al. 1985; Kohn and Movshon 2003; Born and Bradley 2005). Scotopic visual stimulation produces a robust activation in the MT + complex in humans (Hadjikhani and Tootell 2000), which might be rod-biased (Purpura et al. 1988). From an ecological perspective, motion information is important for insect foraging in nocturnal primates (Siemers et al. 2007). Indeed, night monkeys’ diet is more reliant on insects compared to close diurnal relatives (Wright 1989; Fernandez-Duque 2003; Wolovich et al. 2010). Taken together, these studies are consistent with the view that sensory receptors, sensory systems, behavior, and habitat choice are evolutionary coupled (Endler 1992).

Furthermore, night monkey MT neurons are also reported to exhibit distinctive features in comparison to their diurnal NHP relatives, such as object orientation and shape selectivity (Zeki 1980; Malonek et al. 1994), which might be associated with their enhanced sensitivity to temporal and spatial contrast in scotopic conditions (Jacobs 1977a; Jacobs et al. 1979). The relative size of primary visual cortex (V1) in night monkeys was smaller than in marmosets (Fig. 4D), while the relative size of the MT + complex to V1 was significantly larger in night monkeys than in macaques and marmosets (Fig. S3). These results suggest that the expansion is specific to the MT + complex rather than a general expansion of the whole visual system. Indeed, MT receives multiple streams of lower-level visual information directly from subcortical structures (Berman and Wurtz 2010; 2011; Warner et al. 2015), and may contribute to residual visual capacity after V1 lesions (Rodman et al. 1989; Girard et al. 1992; Rosa et al. 2000; Warner et al. 2015; Kato et al. 2021). Therefore, expansion of the MT + complex in night monkeys may be related to the nocturnal adaptation specialized to motion perception independently from the striate visual pathway (Krubitzer and Kaas 1990).

In contrast to the disproportionately large eyes, the term ‘Aotus’ reflects the earless appearance of this genus, with small external ears mostly hidden beneath the fur (Wright 1989). Despite the underdeveloped external auditory organs, our analysis suggests that night monkeys might have a larger auditory cortex relative to the total cortex in comparison to diurnal primates (Fig. 3–F, Fig. 4B). Similar expansion of the auditory cortex was reported in nocturnal rodents (Campi and Krubitzer 2010), suggesting that this might be related to nocturnal adaptation. This expansion might also be related to improved hearing ability, which is important in a nocturnal environment (Kronfeld-Schor and Dayan 2003). However, the auditory sensitivity and frequency range of night monkeys are not significantly different from those of diurnal primates (Beecher 1974; Coleman and Ross 2004). Alternatively, the expansion of auditory cortex may compensate for the reduced visual information with multi-modal integration (Ernst and Bülthoff 2004), as it is known that auditory information can improve visual detection at both neuronal (Meredith and Stein 1986) and behavioral levels (McDonald et al. 2000; Frassinetti et al. 2002). Another hypothesis is that the relatively large auditory cortex may be associated with the evolution of acoustic communication in the nocturnal environment, which may be more effective than visual communication under dim light conditions (Endler 1992; Endler and Basolo 1998; Kronfeld-Schor and Dayan 2003; Chen and Wiens 2020). Indeed, the auditory cortex of night monkeys encodes sounds well matched to the natural conspecific vocalizations (Atencio et al. 2007). Further behavioral and neurobiological studies are needed to elucidate the functional relevance to the expansion of the auditory cortex.

### Expansion of inferior parietal association cortex in primates

Dorso-medial to the MT + complex and the auditory cortex lies an island area of low T1w/T2w myelin in all three species (Fig. 3A–I, cyan border). This region in macaque monkeys corresponds to area 7a, 7b, and 7op (Lewis and Van Essen 2000a), which are closely overlapped with classic Brodmann area 7 (BA7). In marmosets, this region contains the ventral part of the posterior parietal cortex (PPv), which is subdivided into TPt, PF, PFG, PG, and OPt (Rosa et al. 2009; Paxinos et al. 2012). However, little is known about this region in night monkeys, possibly due to its being mostly buried in the lateral sulcus (Fig. 3B) and thus not well characterized in previous studies (Kaas 2004; Sereno et al. 2015). We found that it has low myelin similar to the corresponding region in other NHP species (Fig. 3A, C). In the tissue flatmap of Sereno et al. 2015 (Fig. 3D), this low-myelin area corresponds to the area surrounded by PP/VPP and TA/TD and lacks any annotation. Dorsal to this low myelinated area is a highly myelinated visual area which receives inputs from MT, identified as the lateral intraparietal area (LIP) (Blatt et al. 1990) or its ventral subdivision LIPv (Lewis and Van Essen 2000b) in macaques, ventral posterior parietal area (VPP) in night monkeys (Allman and Kaas 1971a; Krubitzer and Kaas 1993; Sereno et al. 2015), and the dorsal part of posterior parietal cortex (PPd) in marmosets (Palmer and Rosa 2006; Ma et al. 2020). Therefore, accumulated evidence in conjunction with our myeloarchitectonic findings indicates that the low myelinated parietal region preserves its relative position on the cortical surface and likely corresponds to BA7 homologs across three NHP species.

BA7 in NHPs is considered a multi-modal association region contributing to spatial perception, somatosensory, and motor control (Mountcastle et al. 1975; Hyvärinen 1982). The homologous region in humans is considered to be located in the inferior parietal lobule (IPL), primarily based on connectivity studies (Pandya and Seltzer 1982; Caspers et al. 2011, 2013). This area corresponds primarily to Brodmann areas 39/40 and has recently been identified as the PG/PF/PFG complex (Glasser et al. 2016) using the terminology of Von Economo and Koskinas (von Economo and Koskinas 1925). Similar to NHPs, the IPL in humans is involved in spatial perception (Corbetta and Shulman 2002), action perception (Passingham et al. 2014), social cognition (Bzdok et al. 2012, 2016), use of tools (Johnson-Frey et al. 2005; Ramayya et al. 2010), and language (Binder et al. 2009). Comparisons of macaque BA7 and human IPL suggest an evolutionary expansion (Van Essen and Dierker 2007; Xu et al. 2020) or areal duplication and divergence (Yokoyama et al. 2021) of this region.

It is noteworthy that we found evidence for an expansion of BA7 that parallels the expansion of brain size in NHPs (Fig. 4C), suggesting that multi-modal information perception is important across NHPs, but especially in the gyrencephalic macaque. In particular, spatial perception and memory are among fundamental cognitive processes for foraging behaviors and survival of species, possibly relying on the ability to use perceived cues that relate objects or environmental traits to probability of finding food in the decision-making process. While night monkeys may need to be more sensitive to auditory and motion perception in the dim illumination, it is interesting that nocturnal monkeys exhibit similar efficiency in the use of spatial memory (e.g., perceived probability of food location) to diurnal monkeys (Bicca-Marques and Garber 2004). Indeed, experimental studies reveal that the night monkeys’ foraging behavior follows the ‘routes strategy’ rather than unimodal sensory inputs (Bolen and Freen 1997; da Costa and Bicca-Marques 2014). The routes or travel paths to reach food may rely on integrated perceptions of scent marking, visual and auditory cues (Wright 1989), and ecological burden (Rosati 2017). The travel paths of primates in natural environments are being studied to infer spatial cognitive strategies for foraging behaviors under ecological complexity, and primate brain evolution (Janmaat et al. 2021). The foraging behavior under ecological burden is likely associated with evolution of the brain size in primates (DeCasien et al. 2017).

### T1w/T2w MRI-based myeloarchitecture analysis

The cortical T1w/T2w ratio was originally proposed by Glasser and Van Essen as a marker of cortical myelin density (Glasser and Van Essen 2011; Glasser et al. 2014). Mapping of the cortical T1w/T2w ratio correlates well with the cortical-surface maps of myelin staining in humans in the seminal work of Adolph Hopf (Nieuwenhuys 2013). In HCP data, the T1w and T2w images are acquired with high-resolution, 0.8 mm isotropic or better, which corresponds to at least two voxels at the minimum cortical thickness in humans (1.6 mm) (Glasser et al. 2013). To generate T1w/T2w myelin contrast, the T1w and T2w images are carefully registered to each other, and their ratio mapped onto the mid-thickness surface by minimizing partial volume effects, and corrected for MRI-based intensity bias in a spatially low-frequency range. The HCP-style data acquisition and analysis methodology was previously applied to investigate T1w/T2w myeloarchitecture in several NHP species (Van Essen et al. 2019; Hayashi et al. 2021). Species-specific low-frequency bias correction of the T1w/T2w ratio was also applied by taking into account the difference in brain size of NHP including macaques and marmosets (Hayashi et al. 2021; Ose et al. 2022).

The current study applied the same HCP-NHP style approach to the night monkeys, demonstrating that cortical T1w/T2w myelin contrast (Fig. 2B) shows a similar spatial pattern as in other primates (Van Essen et al. 2019; Hayashi et al. 2021) and replicates histological myelin stain results in the parietal and occipital areas of cerebral cortex (Fig. 2D, E). Myelin gradients are in principle insensitive to residual low-frequency biases of myelin maps, allowing us to define gradient-based boundaries of three cortical areas, MT + , auditory, and BA7 semi-automatically across three NHP species. The areal sizes based on our T1w/T2w myelin gradient are comparable with those found in previous histological studies (Table 1, Table S2). It should be noted that our estimate of the MT + complex (47.9 ± 2.3 mm^2^) is larger than a previous report of night monkey MT area (37 mm^2^) (Tootell et al. 1985), probably because their analysis excluded the highly myelinated rostral region corresponding to MST. We applied the same criteria to all three species for a quantitative interspecies comparison (Fig. 3G–I). However, there are a few caveats to consider. First, although the surface area and cortical volume were estimated in the naive physical space of each individual to reflect size differences across subjects, we likely underestimated intersubject variability, because we applied the group average areal boundary based on the average T1w/T2w myelin gradient. Indeed, the areal size displayed larger variability if we defined boundaries based on individual T1w/T2w myelin gradients for each subject separately, which likely reflects a combination of genuine intersubject variability of functional parcellation, but also fluctuations related to noisier data (Fig. S4). Second, the boundaries of MT + complex, auditory cortex, and BA7 do not precisely match published parcellations (e.g., Lewis and Van Essen 2000a for macaques, Paxinos et al. 2012 for marmosets). The current method mainly focused on myeloarchitecture, but will likely benefit from information from cytoarchitecture, connectivity, and function for more accurate parcellations. Third, sampling differences, particularly in age, might have biased our results, as all of our night monkeys were older than any of the macaques or marmosets (Table S1). These sampling differences are largely due to the limited availability of NHP animals for experimental use. However, we reduced effects of age using T1w/T2w myelin gradient, which is less sensitive to low-frequency spatial information such as the age effects on T1w/T2w myelin reported in humans (Baum et al. 2022; Grydeland et al. 2013). A detailed analysis of age and sex effects would be beneficial for intra- and interspecies comparisons. Despite these limitations, our methodology enables non-invasive and quantitative comparisons across NHP species. The overall results indicate that our parieto-temporal parcellation based on T1w/T2w myelin contrast showed reasonable estimates across NHP species, providing a valuable basis for interspecies comparisons. Multimodal surface matching including the T1w/T2w myelin map and/or functional connectivity would be useful for more detailed percellation of areas including those with lower myelination (Glasser et al. 2016), and should be addressed in future studies.

## Supplementary Information

Below is the link to the electronic supplementary material.

Supplementary file1 (DOCX 6315 KB)

## Data Availability

Data are partly available at BALSA https://balsa.wustl.edu/. Analysis pipeline is available at https://github.com/Washington-University/NHPPipelines. Protocols are available at https://brainminds-beyond.riken.jp. Additional data are available from corresponding author upon request.
