# Supplementary material for: Cortical adaptation of the night monkey to a nocturnal niche environment: a comparative non-invasive T1w/T2w myelin study
Source: Brain Struct Funct. 2022 Nov 18;228(5):1107–23. doi: 10.1007/s00429-022-02591-x (PMC10192470; doi:10.1007/s00429-022-02591-x)
Supplement: Supplementary file 1 — Supplementary file1 (DOCX 6315 KB) [file 429_2022_2591_MOESM1_ESM.docx]

## **Supplementary Information**

**Cortical adaptation of the night monkey to a nocturnal niche environment: A comparative non-invasive T1w/T2w myelin study**

**Authors**

Takuro Ikeda^1^, Joonas A. Autio^1^, Akihiro Kawasaki^1^, Chiho Takeda^1^, Takayuki Ose^1^, Masahiko Takada^2^, David C. Van Essen^3^, Matthew F. Glasser^3,4^, Takuya Hayashi^1^

**Affiliations**

^1^Laboratory for Brain Connectomics Imaging, RIKEN Center for Biosystems Dynamics Research, Kobe, Japan

^2^Center for the Evolutionary Origins of Human Behavior, Kyoto University, Inuyama, Japan

^3^Department of Neuroscience, Washington University Medical School, St Louis, MO\

^4^Department of Radiology, Washington University Medical School, St Louis, MO

**Incl.**

Figure S1, S2, S3, S4,

Table S1, S2


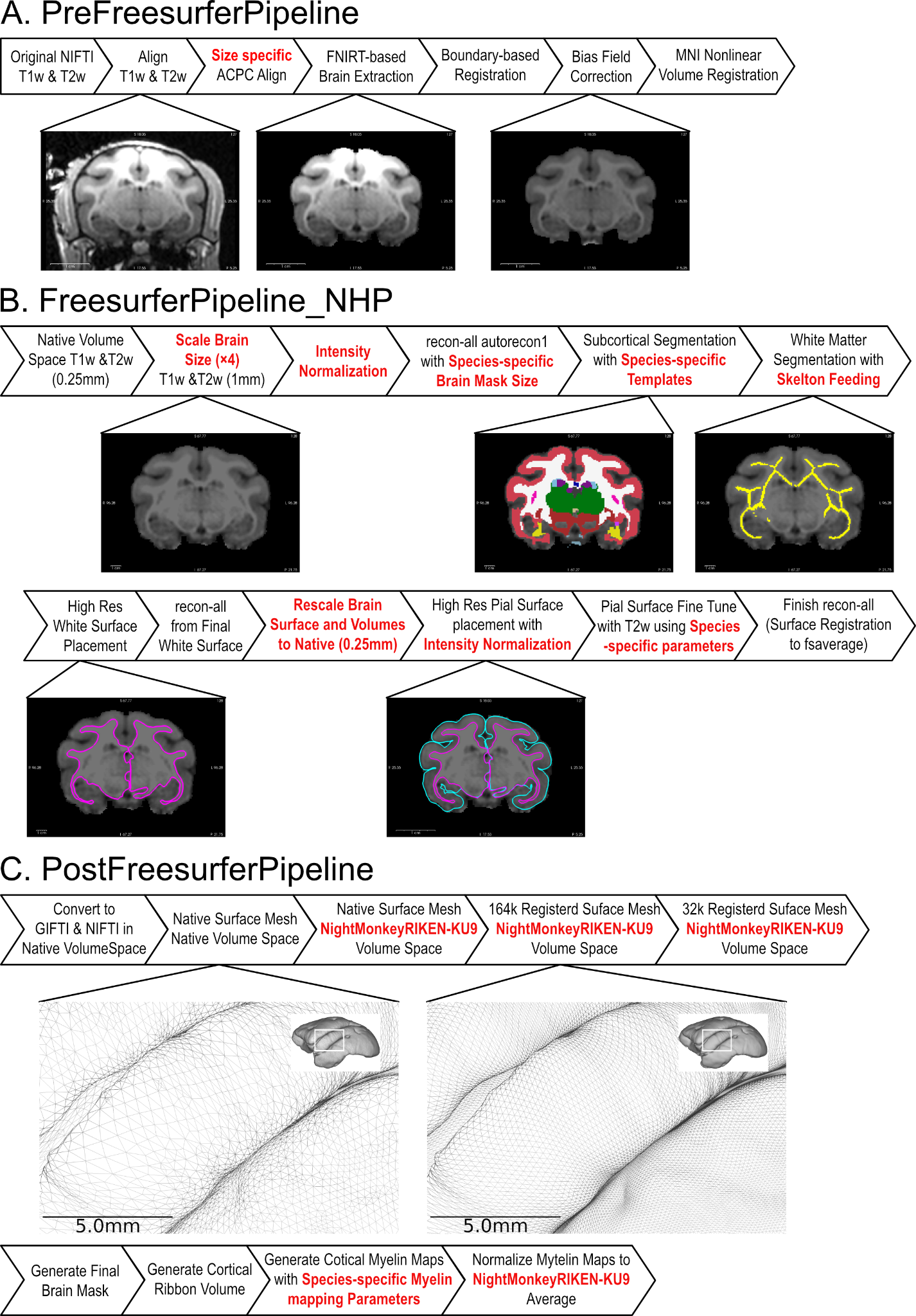


**Supplementary Figure S1. HCP-NHP pipeline adapted for structural image processing in night monkeys.**

An overview of the non-human primate (NHP) version of the HCP pipeline consists of **(A)** PreFreeSurfer Pipeline, **(B)** FreeSurfer Pipeline, and **(C)** PostFreeSurfer Pipeline. Texts in red describe adaptations of the pipeline to the night monkey brain. Preprocessed T1w images in a representative subject (NM52) are shown at each step.

###
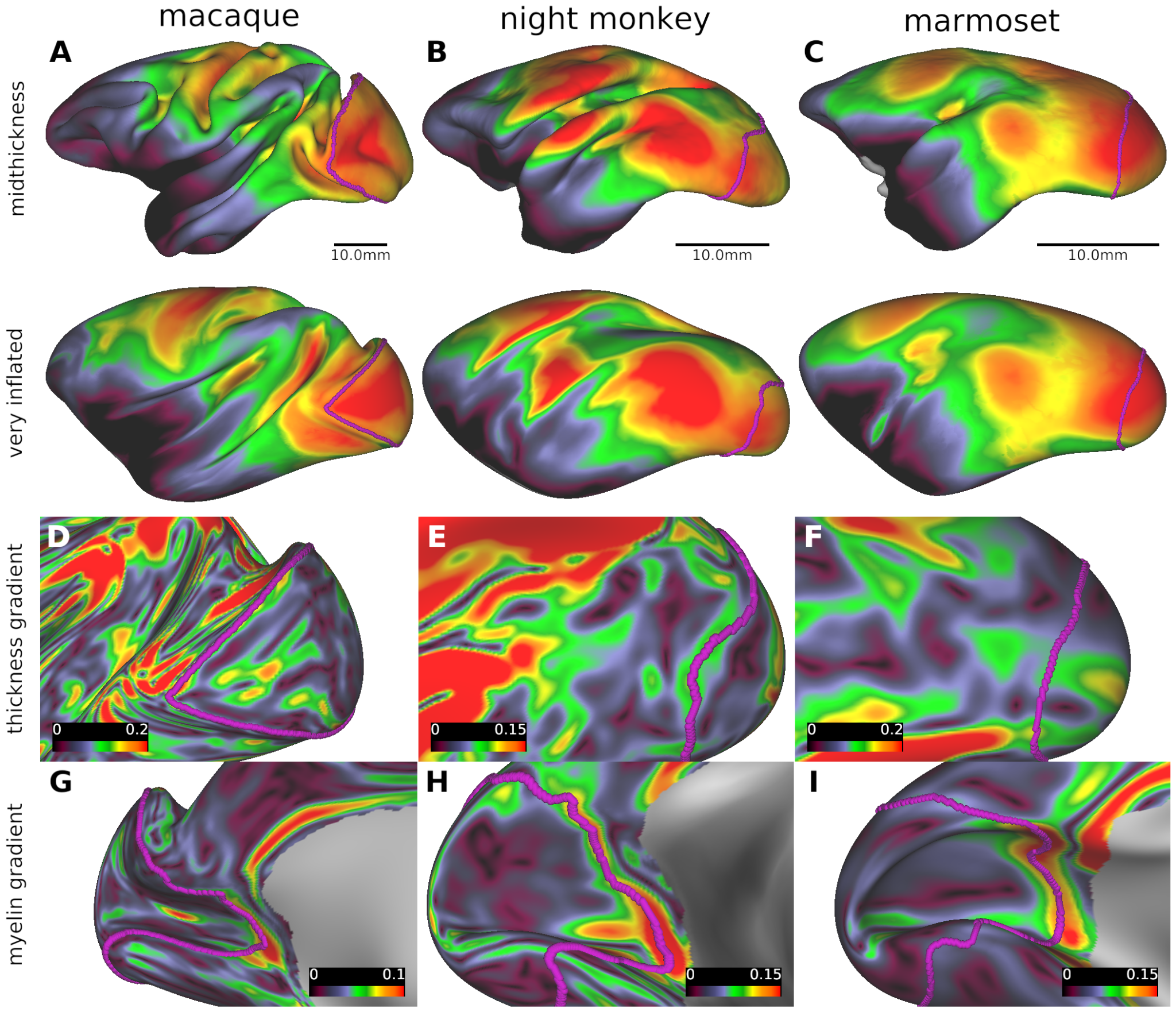


**Supplementary Figure S2.** **Interspecies comparison of primary visual cortex (V1).**

T1w/T2w myelin contrast in **(A)** macaque, **(B)** night, and **(C)** marmoset monkeys displayed on a midthickness surface (top row) and a very inflated surface (bottom row) with V1 border (purple line). V1 borders were defined based on thickness gradient (lateral side; **D, E, F**) and myelin gradient (medial side**; G, H, I**).

###
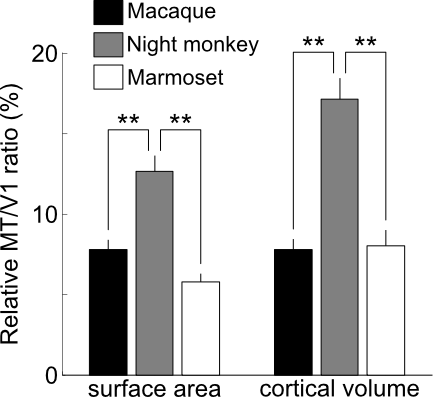


**Supplementary Figure S3.** **Relative size of MT in comparison to V1.**

The error-bars indicate the standard deviation across subjects (macaque N=32x2, night monkey N=9x2, marmoset N=20x2). Interspecies differences were tested by 1-way ANOVA, followed by t-test with Bonferroni correction. * and ** indicate corrected *p* < 0.05 and 0.001, respectively.


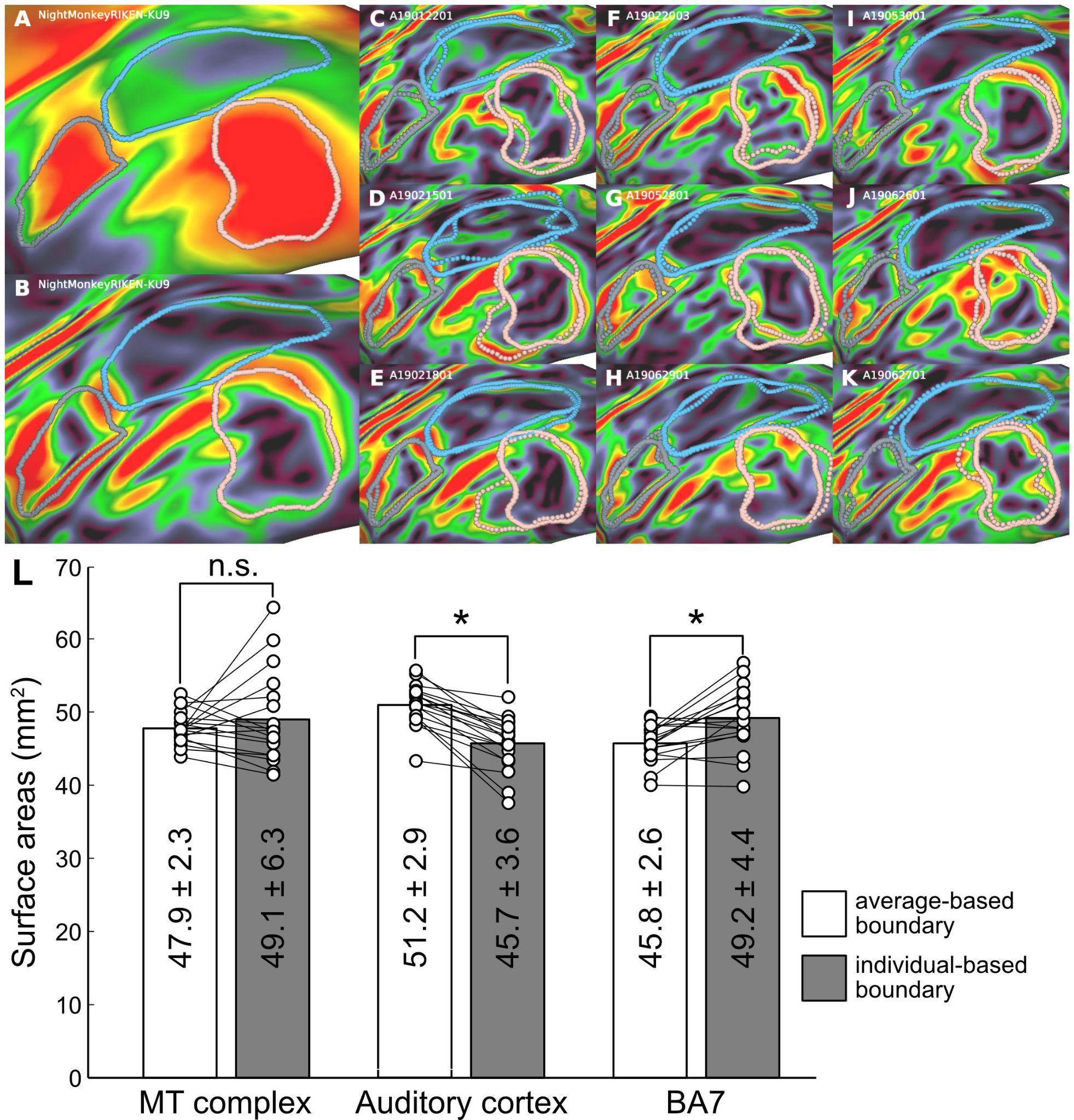


**Supplementary Figure S4.** **Inter-subject comparison of myeloarchitecture in parieto-temporal cortex of night monkey.**

**(A)** Average T1w/T2w myelin contrast **(B)** and its gradient in night monkey (NightMonkeyRIKEN-KU9). The areal borders of MT+ complex (pink), auditory cortex (gray), and area 7 (cyan) were defined based on average T1w/T2w myelin gradient. **(C-K)** Individual T1w/T2w myelin gradients (N=9). Solid lines indicate the areal borders based on the average gradient while dotted lines indicate the areal borders based on the individual gradients. **(L)** Comparisons of surface area determined using average-based boundary (white) and individual-based boundary (gray), with mean ± SD inside the bars. * indicates *p* < 0.05 by paired t-test (N=9x2).

| Species | Macaque | | Night monkey | Marmoset |
| --- | --- | --- | --- | --- |
|  | *Macaca mulatta* | *Macaca fascicularis* | *Aotus lemurinus* | *Callithrix jacchus* |
| *N*, sex | 18 males, 4 females | 10 males | 4 males, 5 females | 20 males |
| age (y.o.) | 5.3 ± 1.7 | 5.4 ± 2.4 | 24.2 ± 6.7 | 5.5 ± 2.8 |
| body weight. (kg) | 5.20 ± 1.33 | 4.51 ± 1.50 | 1.10 ± 0.07 | 0.38 ± 0.06 |
| scanner | MAGNETOM Prisma, Siemens, 3T | | | |
| # of channel of RF coil | 24 | | | 16 |
| spatial resolution (mm^3^) | 0.5×0.5×0.5 | 0.5×0.5×0.5 | 0.25×0.25×0.5 | 0.36×0.36×0.36 |
| T1w sequence | MPRAGE | | | |
| TR/TE/TI (ms) | 2200/2.2/900 | 2200/2.2/900 | 2200/2.2/900 | 2200/2.58/700 |
| FOV (mm) | 128×128×112 | 128×128×112 | 128×128×112 | 70×59×46 |
| T2w sequence | T2-SPACE | | | |
| TR/TE (ms) | 3200/562 | 3200/562 | 3000/562 | 3000/558 |
| FOV (mm) | 128×128×112 | 128×128×112 | 128×128×112 | 70×59×46 |

**Supplementary Table S1.**

Population characteristics and experimental conditions

| Species | Cortical parcel of interest | Cortical volume (mm^3^) (N: number of hemispheres investigated) | Methods | Reference |
| --- | --- | --- | --- | --- |
| Marmoset | MT+ complex | 24.2 ± 3.7 (N=40) | T1w/T2w myelin | Current study |
|  |  | 31.56 (N = 3) | 3D reconstruction of postmortem brain, parcellation based on Paxinos et al. 2012 (MT+MST) | (Atapour et al. 2019) |
|  | V1 | 303.0 ± 23.0 (N=40) | T1w/T2w myelin | Current study |
|  |  | 298.13 (N=3) | 3D reconstruction of postmortem brain, parcellation based on Paxinos et al. 2012 | (Atapour et al. 2019) |
|  | Auditory cortex | 26.7 ± 3.2 (N=40) | T1w/T2w myelin | Current study |
|  |  | 22.31 (N=3) | 3D reconstruction of postmortem brain, parcellation based on Paxinos et al. 2012 (A1, R, CM, CL) | (Atapour et al. 2019) |

**Supplementary Table S2.**

Comparisons of the cortical volume of MT+ complex, V1, and auditory cortex in marmoset. Brain shrinkage was corrected using a shrinkage factor of 0.801 in Atapour et al. 2019.

Atapour N, Majka P, Wolkowicz IH, et al (2019) Neuronal Distribution Across the Cerebral Cortex of the Marmoset Monkey (Callithrix jacchus). Cerebral Cortex 29:3836–3863. https://doi.org/10.1093/cercor/bhy263
